# Efficacy of Eye-Movement Desensitization and Reprocessing for Patients with Posttraumatic-Stress Disorder: A Meta-Analysis of Randomized Controlled Trials

**DOI:** 10.1371/journal.pone.0103676

**Published:** 2014-08-07

**Authors:** Ying-Ren Chen, Kuo-Wei Hung, Jui-Chen Tsai, Hsin Chu, Min-Huey Chung, Su-Ru Chen, Yuan-Mei Liao, Keng-Liang Ou, Yue-Cune Chang, Kuei-Ru Chou

**Affiliations:** 1 Graduate Institute of Nursing, College of Nursing, Taipei Medical University, Taipei, Taiwan, and Taoyuan Armed Forces General Hospital, Longtan, Taiwan; 2 Division of Neurology, Department of Internal Medicine, Yuan's General Hospital, Kaohsiung, Taiwan; 3 Department of Nursing, Taipei Medical University-Shuang Ho Hospital, Taipei, Taiwan; 4 Institute of Aerospace and Undersea Medicine, School of Medicine, National Defense Medical Center, Taipei, Taiwan; 5 Department of Neurology, Tri-Service General Hospital, National Defense Medical Center, Taipei, Taiwan; 6 School of Nursing, College of Nursing, Taipei Medical University, Taipei, Taiwan; 7 Graduate Institute of Biomedical Materials and Tissue Engineering, College of Oral Medicine, Taipei Medical University, Taipei, Taiwan; 8 Research Center for Biomedical Devices and Prototyping Production, Taipei Medical University, Taipei, Taiwan; 9 Research Center for Biomedical Implants and Microsurgery Devices, Taipei Medical University, Taipei, Taiwan; 10 Department of Dentistry, Taipei Medical University-Shuang-Ho Hospital, Taipei, Taiwan; 11 Department of Mathematics, Tamkang University, Tamsui, Taiwan; 12 Psychiatric Research Center, Taipei Medical University Hospital, Taipei, Taiwan; University of California, San Francisco, United States of America

## Abstract

**Background:**

We performed the first meta-analysis of clinical studies by investigating the effects of eye-movement desensitization and reprocessing (EMDR) therapy on the symptoms of posttraumatic stress disorder (PTSD), depression, anxiety, and subjective distress in PTSD patients treated during the past 2 decades.

**Methods:**

We performed a quantitative meta-analysis on the findings of 26 randomized controlled trials of EMDR therapy for PTSD published between 1991 and 2013, which were identified through the ISI Web of Science, Embase, Cochrane Library, MEDLINE, PubMed, Scopus, PsycINFO, and the Cumulative Index to Nursing and Allied Health Literature electronic databases, among which 22, 20, 16, and 11 of the studies assessed the effects of EMDR on the symptoms of PTSD, depression, anxiety, and subjective distress, respectively, as the primary clinical outcome.

**Results:**

The meta-analysis revealed that the EMDR treatments significantly reduced the symptoms of PTSD (*g* = −0.662; 95% confidence interval (CI): −0.887 to −0.436), depression (*g* = −0.643; 95% CI: −0.864 to −0.422), anxiety (*g* = −0.640; 95% CI: −0.890 to −0.390), and subjective distress (*g* = −0.956; 95% CI: −1.388 to −0.525) in PTSD patients.

**Conclusion:**

This study confirmed that EMDR therapy significantly reduces the symptoms of PTSD, depression, anxiety, and subjective distress in PTSD patients. The subgroup analysis indicated that a treatment duration of more than 60 min per session was a major contributing factor in the amelioration of anxiety and depression, and that a therapist with experience in conducting PTSD group therapy was a major contributing factor in the reduction of PTSD symptoms.

## Introduction

Eye movement desensitization and reprocessing (EMDR) was developed by Francine Shapiro [1], and is a complex and specific desensitizing treatment method. EMDR therapy desensitizes patients to anxiety and integrates information processing [1]. Adaptive information processing is the theoretical framework for EMDR, because it addresses factors related to both pathology and personality development. Adaptive information processing contributes to orienting responses (ORs), which involve retrieving information from previous experiences and integrating them into a positive emotional and cognitive schema [2]. A dual-attention stimulus, such as eye movement, is an integral component of EMDR because it induces certain physiological conditions that activate information processing. Eye movements may unblock the information-processing centers of the brain, creating a connection between stored information on previous events and adverse outcomes that is used to generate a response to a current stimulus. Subsequently aroused relaxation responses or a new series of physiological responses reconnect to the stored information on previous adverse experiences, and the new information is reintegrated [2].

A meta-analysis was conducted by Davidson and Parker [3] to analyze 34 controlled experimental studies that had examined the effects of EMDR therapy on patients with anxiety disorders. Their results indicated that EMDR therapy significantly reduced the symptoms of anxiety disorders, with a Cohen's *d* of 0.87 and a 95% confidence interval (CI) of 0.18 to 0.58 (*p*<0.01). Another meta-analysis of controlled experimental studies conducted by Rodenburg et al. [4] revealed that EMDR therapy significantly reduced the symptoms of posttraumatic stress disorder (PTSD) in children, with a Cohen's *d* of 0.56 and a 95% CI of 0.42 to 0.70 (*p*<0.001).

A literature review reported that EMDR therapy significantly reduced the symptoms of depression, thereby reducing the Montgomery-Asberg Depression Rating Scale (MADRS), Hamilton Depression Scale (HAM-D), and Beck Depression Inventory (BDI) scores from 26.4 to 9.3, 29.5 to 26.8, and 25.95 to 10.70, respectively [5]–[7]. Previous studies have indicated that EMDR therapy significantly reduced the symptoms of anxiety, reducing the Beck Anxiety Inventory (BAI) and State-Trait Anxiety Inventory (STATE) scores from 33.8 to 16.2 and 51.10 to 32.60, respectively, and similar studies of anxiety reported that EMDR therapy reduced the Hamilton Anxiety Scale (HAM-A), Hospital Anxiety and Depression Scale (HADS), and STATE scores from 26.2 to 9.1, 15.3 to 7.7, and 52.14 to 35.17, respectively, among which one study reported a moderate effect size and a Cohen's *d* of 0.66 [6]–[9]. Other previous studies have indicated that EMDR significantly improved the subjective distress index, with Cohen's *d* ranging from 1.04 to 2.07 and the mean subjective units of distress (SUD) reduced from 7.02 to 2.72 [8], [10]–[16].

Despite the wealth of information on the efficacy of EMDR for treating PTSD, the magnitude of the effects of EMDR therapy on anxiety, depression, and subjective distress in PTSD patients remains largely unclear. We performed a quantitative meta-analysis on the findings of various clinical studies reported between 1991 and 2013 that have investigated the effects of EMDR therapy on the symptoms of PTSD, depression, anxiety, or subjective distress in PTSD patients. Our results indicated that EMDR therapy significantly reduced the symptoms of PTSD, depression, anxiety, and subjective distress in PTSD patients, with moderate to large effect sizes.

## Materials and Methods

### Reporting Standards

The current study was conceived, conducted, and reported according to the Preferred Reporting Items for Systematic Reviews and Meta-analyses (PRISMA) statement for meta-analyses of randomized controlled trials (RCTs).

### Search Strategy

This study included a quantitative investigation of studies involving the use of EMDR for treating PTSD that were published between January 1991 and December 2013, which were identified using the ISI Web of Science, Embase, Cochrane Library, MEDLINE, PubMed, SCOPUS, PsycINFO, and Cumulative Index to Nursing and Allied Health Literature electronic databases. Studies were identified through database searches conducted using the medical subject headings (MeSHs) “eye movement desensitization reprocessing” and “posttraumatic stress disorder”, or keyword searches using “PTSD” and “EMDR” or “eye movement desensitization.” The Web sites of professional associations and the reference lists of relevant articles were examined, and Internet searches were performed using the Google Scholar search engine to identify additional studies that had not yet been included in the aforementioned electronic databases.

### Inclusion and Exclusion Criteria

**Figure 1:**
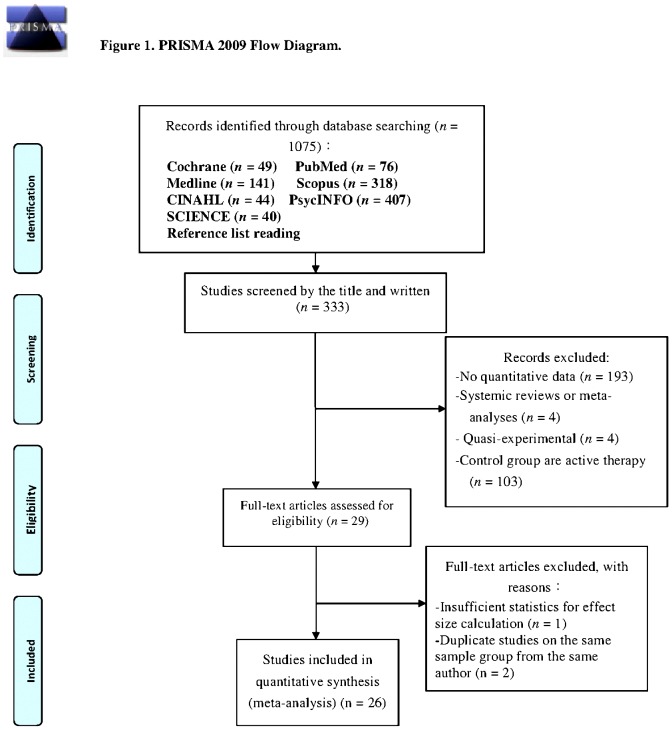
PRISMA 2009 flow diagram.

The inclusion criteria for the current study were based on those used in similar studies and our research objectives. Previous studies were selected for the meta-analysis based on the following inclusion criteria: (1) published between January 1991 and December 2013; (2) included PTSD patients treated with EMDR; (3) met the requirements of an RCT established by the Cochrane Collaboration [17]; (4) EMDR was administered by trained professionals, including physicians, nurses, or psychotherapists; (5) control patients received other treatment or no treatment; and (6) the assessment of clinical outcomes included an adequate statistical analysis of the effect size, such as the mean, standard deviation, mean difference, sample size, *t* value, *F* value, odds ratio (OR), or P value. Duplicate publications, qualitative studies, quasi-experimental studies, and single-subject or single-group experimental studies were excluded. The RCT selection process is depicted in Figure 1.

### Outcome Measures

We considered various clinical outcomes that were reported in the selected RCTs to demonstrate improvements in the symptoms of PTSD, depression, anxiety, and subjective distress. PTSD symptoms were assessed using the Clinician-Administered PTSD Scale (CAPS), the PTSD Checklist (PCL-C), the Child Report of Posttraumatic Symptoms (CROPS), the self-reported Symptom Checklist of the Structured Interview for PTSD (SI-PTSD), and the Impact of Event Scale (IES). Depression was assessed using the HADS, MADRS, BDI, and HAM-D instruments. Anxiety was assessed using the HAM-A, BAI, and STATE instruments. Subjective distress was assessed based on the SUD instrument.

### Data Extraction

Sample selection and variable interpretation are easily biased. Therefore, Cohen's κ was used to evaluate the reliability between raters and registrants to avoid bias associated with sample selection or variable interpretation, with a value of κ>0.65 indicating acceptable consistency between raters and registrants [18]. A doctoral student experienced in psychiatric studies and trained in research methodology registered the clinical variables. The investigator and the collaborative rater separately registered all of the selected studies with regard to design, diagnosis, intervention, interveners, and outcome variables, and both performed an inter-rater reliability test, yielding a kappa value of 0.86. If disagreements occurred between the two reviewers, a third reviewer, a professor with experience performing meta-analyses, reconciled the difference. We contacted several of the authors directly and obtained clarification regarding data that were not included in the published report. The results of the analysis of the outcome measures of the selected studies are shown in Table 1.

**Table 1:** Characteristics of EMDR for patients with PTSD of the randomized controlled trials (RCTs) included in the meta-analysis (*N* = 26).

Study	Intervention	Design	Sample size(patients)	Intervention characterization	Outcome	Study quality/
	(Experimental/Control)					Cochrane tool
Vaughan et al. (1994)	EMD/AMR	RCT	Total N: 36	Number of times treated: 4	**self-rating scales**	8
			Complete N (T/C): 12/11	Treatment duration (min): 50	Anxiety (SYAI)	AA:low
			Mean age (yr): 32	Each group size (people): individual	Depression (BDI)	AC:unclear
				EMDR therapist: -	**non-self-rating scales**	BAO:low
				PTSD group experience: undisclosed	Symptoms (SI-PTSD)	IO:low
					Depression (HRSD)	SRO:low
Jensen (1994)	MDR/delayed MDR	RCT	Total N: 25	Number of times treated: 3	**self-rating scales**	6/
			Complete N (T/C): 13/12	Treatment duration (min): -	SUD	AA:low
			Mean age (yr): 43.1	Each group size (people): individual	**non-self-rating scales**	AC:unclear
				EMDR therapist: -	Symptoms (SI-PTSD)	BAO:unclear
				PTSD group experience: yes		IO:high
						SRO:low
Wilson et al. (1995)	EMDR/delayed MDR	RCT	Total N: 80	Number of times treated: 3	**self-rating scales**	8/
			Complete N (T/C): -/-	Treatment duration (min): 90	Symptoms (IES)	AA:low
			Mean age (yr): 39	Each group size (people): individual	Anxiety (STAI-state/trait)	AC:unclear
				EMDR therapist: certified professional	Depression (SCL-R-D)	BAO:unclear
				PTSD group experience: yes		IO:unclear
						SRO:low
Dunn et al. (1996)	EMDR/visual placebo	RCT	Total N: 28	Number of times treated: -	**self-rating scales**	6/
			Complete N (T/C): -/-	Treatment duration (min): -	SUD	AA:low
			Mean age (yr):-	Each group size (people): individual		AC:unclear
				EMDR therapist: certified professional		BAO:unclear
				PTSD group experience: yes		IO:low
						SRO:low
Rothbaum (1997)	EMDR/WL	RCT	Total N: 18	Number of times treated: 3	**self-rating scales**	7/
			Complete N (T/C): 10/8	Treatment duration (min): 90	Symptoms (IES)	AA:low
			Mean age (yr): 34.6	Each group size (people): individual	Depression (BDI)	AC:unclear
				EMDR therapist: certified professional	Anxiety (STAI-state/trait)	BAO:low
				PTSD group experience: yes	**non-self-rating scales**	IO:high
					Symptoms(PTSD-symptoms, PSS)	SRO:low
Marcus et al. (1997)	EMDR/SC	RCT	Total N: 67	Number of times treated: 3	**self-rating scales**	7/
			Complete N (T/C): -/-	Treatment duration (min): 50	Symptoms (Modified PTSD scale)	AA:low
			Mean age (yr): 42.4	Each group size (people): individual	Symptoms (IES)	AC:unclear
				EMDR therapist: nil	Anxiety (STAI-state/trait)	BAO:low
				PTSD group experience: yes	SUD	IO:unclear
						SRO:low
Devilly et al. (1998)	EMDR/SPS	RCT	Total N: 51	Number of times treated: 2	**self-rating scales**	8/
			Complete N (T/C): 20/10	Treatment duration (min): 90	Symptoms (M-PTSD)	AA:low
			Mean age (yr): 50.1	Each group size (people): individual	Anxiety (STAI-Y2)	AC:unclear
				EMDR therapist: certified professional	SUD	BAO:unclear
				PTSD group experience: yes		IO:high
						SRO:low
Scheck et al. (1998)	EMDR/AL	RCT	Total N: 60	Number of times treated: 2	self-rating scales	7/
			Complete N (T/C): 30/30	Treatment duration (min): 90	Symptoms (IES) (PENN)	AA:low
			Mean age (yr): 20.9	Each group size (people): individual	Depression (BDI)	AC:low
				EMDR therapist: certified professional	Anxiety (STAI-state)	BAO:low
				PTSD group experience: yes		IO:high
						SRO:low
Carlson et al. (1998)	EMDR/routine clinical care	RCT	Total N: 35	Number of times treated: 12	**self-rating scales**	8/
			Complete N (T/C): 10/12	Treatment duration (min): 60∼75	Symptoms (PTSD-symptoms)	AA:low
			Mean age (yr): 48.3	Each group size (people): individual	Symptoms (IES)	AC:unclear
				EMDR therapist: nil	Anxiety(STAI-state/trait)	BAO:low
				PTSD group experience: yes	Depression (BDI)	IO:low
						SRO:low
						
Rogers et al. (1999)	EMDR/exposure	RCT	Total N: 12	Number of times treated: 1	**self-rating scales**	6/
			Complete N (T/C): 6/6	Treatment duration (min): 60∼90	Symptoms (IES)	AA:low
				Each group size (people): individual	SUD	AC:unclear
			Mean age (yr): 47∼53	EMDR therapist: -		BAO:low
				PTSD group experience: yes		IO:low
						SRO:low
Devilly & Spence (1999)	EMDR/TTP	RCT	Total N: 23	Number of times treated: 8	**self-rating scales**	7/
			Complete N (T/C): 11/12	Treatment duration (min): 90∼120	Symptoms (IES) (PSS-SR)	AA:low
			Mean age (yr): 38	Each group size (people): individual	Anxiety (STAI-trait)	AC:unclear
				EMDR therapist: -	Depression (BDI)	BAO:unclear
				PTSD group experience: yes		IO:low
						SRO:low
Wilson et al. (2001)	EMDR/SMP	RCT	Total N: 62	Number of times treated: 3	**self-rating scales**	7/
			Complete N (T/C): 33/29	Treatment duration (min): -	SUD	AA:low
			Mean age (yr): 36.8	Each group size (people): individual	**non-self-rating scales**	AC:unclear
				EMDR therapist: -	Symptom (PTSD-symptoms)	BAO:low
				PTSD group experience: disclosed		IO:high
						SRO:low
Power et al. (2002)	EMDR/WL	RCT	Total N: 72	Number of times treated: 1	**self-rating scales**	8/
			Complete N(T/C): 27/24	Treatment duration (min): 90	Symptoms (IOS)	AAA:low
			Mean age (yr): 39.4	Each group size (people): individual	Symptoms (SI-PTSD)	AC:unclear
				EMDR therapist: certified professional	Depression (HADS-D)	BAO:low
				PTSD group experience: yes	Anxiety (HADS-A)	IO:high
						SRO:low
Ironson et al. (2002)	EMDR/PE	RCT	Total N: 22	Number of times treated: 6	**self-rating scales**	7/
			Complete N(T/C): 10/12	Treatment duration (min): 90	Symptoms (PSS-SR)	AA:low
			Mean age (yr): 16∼62	Each group size (people): individual	Depression (BDI)	AC:unclear
				EMDR therapist: certified professional	SUD	BAO:high
				PTSD group experience: yes		IO:high
						SRO:low
Chemtob et al. (2002)	EMDR/WL	RCT	Total N: 32	Number of times treated: 3	**self-rating scales**	7/
			Complete N(T/C): 17/15	Treatment duration (min): -	Depression (CDI)	AA:low
			Mean age (yr): 8.4	Each group size (people): individual	Anxiety (RCMAS)	AC:unclear
				EMDR therapist: certified professional	**non-self-rating scales**	BAO:unclear
				PTSD group experience: yes	Symptoms (CRI)	IO:low
						SRO:low
Lee et al. (2002)	EMDR/SITPE	RCT	Total N: 24	Number of times treated: 7	**self-rating scales**	6/
			Complete N(T/C): 12/12	Treatment duration (min): 90	Symptoms (IES)	AA:low
			Mean age (yr): 35.3	Each group size (people): individual	Depression (BDI)	AC:unclear
				EMDR therapist: certified professional	**non-self-rating scales**	BAO:high
				PTSD group experience: yes	Symptoms (SI-PTSD)	IO:high
						SRO:low
Lytle et al. (2002)	EMDR/non-direct therapy	RCT	Total N: 45	Number of times treated: 1	**self-rating scales**	7/
			Complete N(T/C): 15/15	Treatment duration (min): 60	Symptoms (IES)	AA:low
			Mean age (yr): 18.95	Each group size (people): individual	Depression (BDI)	AC:unclear
				EMDR therapist: -	Anxiety (STAI-trait)	BAO:unclear
				PTSD group experience: yes		IO:low
						SRO:low
Taylor et al. (2003)	EMDR/relaxation training	RCT	Total N: 60	Number of times treated: 8	**self-rating scales**	7/
			Complete N(T/C): 19/19	Treatment duration (min): 90	Depression (BDI)	AA:low
			Mean age (yr): 37	Each group size (people): individual	**non-self-rating scales**	AC:unclear
				EMDR therapist: certified professional	Symptoms (CAPS)	BAO:low
				PTSD group experience: yes		IO:low
						SRO:low
Jaberghaderi et al. (2004)	EMDR/CBT	RCT	Total N: 62	Number of times treated: 12	**self-rating scales**	6/
			Complete N (T/C): 60/60	Treatment duration (min): 30∼45	Symptoms (CROPS)	AA:low
			Mean age (yr): 12∼13	Each group size (people): individual		AC:unclear
				EMDR therapist: certified professional		BAO:low
				PTSD group experience: yes		IO:low
						SRO:low
Rothbaum et al. (2005)	EMDR/WL	RCT	Total N: 60	Number of times treated: 9	**self-rating scales**	8/
			Complete N (T/C): 20/20	Treatment duration (min): 90	Depression (BDI)	AA:low
			Mean age (yr): 33.8	Each group size (people): individual	Symptoms (IES)	AC:unclear
				EMDR therapist: -	Anxiety (STAI-state/trait)	BAO:unclear
				PTSD group experience: disclosed		IO:low
						SRO:low
Van der Kolk et al. (2007)	EMDR/placebo	RCT	Total N: 88	Number of times treated: 6	**self-rating scales**	7/
			Complete N (T/C): 29/29	Treatment duration (min): 90	Depression (BDI-II)	AA:low
			Mean age (yr): 36.1	Each group size (people): individual	**non-self-rating scales**	AC:unclear
				EMDR therapist: certified professional	Symptoms (CAPS)	BAO:low
				PTSD group experience: yes		IO:low
						SRO:low
Hogberg et al. (2007)	EMDR/WL	RCT	Total N: 24	Number of times treated: 5	**self-rating scales**	6/
			Complete N (T/C): 13/11	Treatment duration (min): 90	Symptoms (IES)	AA:low
			Mean age (yr): 43	Each group size (people): individual	Anxiety (BAI)	AC:low
				EMDR therapist: certified professional	**non-self-rating scales**	BAO:unclear
				PTSD group experience: yes	Anxiety (HAMA-A)	IO:low
					Depression (HAMA-D)	SRO:low
Ahmad et al. (2007)	EMDR/WL	RCT	Total N: 33	Number of times treated: 8	**non-self-rating scales**	7/
			Complete N (T/C): 17/16	Treatment duration (min): 45	Symptoms (PTSS-C)	AA:low
			Mean age (yr): 9.95	Each group size (people): individual		AC:unclear
				EMDR therapist: -		BAO:low
				PTSD group experience: disclosed		IO:low
						SRO:low
Abbasnejad et al. (2007)	EMDR/WL	RCT	Total N: 41	Number of times treated: 4	**self-rating scales**	7/
			Complete N (T/C): 21/20	Treatment duration (min): 90	Depression (BDI)	AA:low
			Mean age (yr): -	Each group size (people): individual	Anxiety (BAI)	AC:unclear
				EMDR therapist: nil	SUD	BAO:unclear
				PTSD group experience: yes		IO:low
						SRO:low
Kemp et al. (2009)	EMDR/WL	RCT	Total N: 27	Number of times treated: 4	**self-rating scales**	7/
			Complete N (T/C): 13/14	Treatment duration (min): 60	Depression (CDS)	AA:low
			Mean age (yr): 8.93	Each group size (people): individual	SUD	AC:unclear
				EMDR therapist: certified professional	**non-self-rating scales**	BAO:unclear
				PTSD group experience: yes	Symptoms (Child PTS-RI)	IO:high
						SRO:low
Karatzias et al. (2011)	EMDR/EFT	RCT	Total N: 46	Number of times treated: 8	**self-rating scales**	8/
			Complete N (T/C): 23/23	Treatment duration (min): 60	Symptoms (PCL-C)	AA:low
			Mean age (yr): 40.6	Each group size (people): individual	Depression (HADS-D)	AC:unclear
				EMDR therapist: -	Anxiety (HADS-A)	BAO:low
				PTSD group experience: disclosed	**non-self-rating scales**	IO:high
					Symptoms (CAPS)	SRO:low

Instruments: M-PTSD  =  Mississippi scale for combat-related PTSD; IES  =  Impact of Event Scale; SI-PTSD  =  Davidson's Structured Interview for PTSD; PSS-SR  =  PTSD Symptom Scale, Self-Report; CAPS  =  Clinician-Administered PTSD Scale; PCL-C  =  PTSD Checklist; SR  =  self-report; Child PTS-RI  =  Child Posttraumatic Stress Reaction Index; CROPS  =  Child Report of Posttraumatic Symptoms; PENN  =  Penn Inventory for Posttraumatic Stress Disorder; CRI  =  Children's Reaction Inventory; PTSS-C  =  Posttraumatic Stress Symptom Scale for Children; HRSD =  The 17-item Hamilton Rating Scale for Depression; BDI =  Beck Depression Inventory; HADS-A =  the Hospital Anxiety and Depression Scale –Anxiety; HADS-D =  the Hospital Anxiety and Depression Scale – Depression; MADRS =  The Montgomery–Åsberg Depression Rating Scale; CDI =  The Children's Depression Inventory; HAMA-A =  the Hospital Anxiety and Hamilton Anxiety Scale–Anxiety; HAMA-D =  the Hospital Anxiety and Hamilton Anxiety Scale–Depression; CDS =  Children's Depression Scale; BAI =  Beck Anxiety Inventory; STAI =  State-Trait Anxiety Inventory.

Intervention: AEM  =  automated EMD; AVA  =  active visual attention; IHT  =  image habituation training; AMR  =  applied muscle relaxation; REDDR  =  EMDR treatment minus the eye movements; SPS  =  standard psychiatric support; AL  =  active listening; E + CR  =  exposure combined with cognitive restructuring; PE  =  prolonged exposure; SITPE  =  stress inoculation training with prolonged exposure; EFD  =  eye fixation desensitization; CBT  =  cognitive behavioral therapy; EFT  =  emotional freedom techniques; SC  =  standard care; WL = : waiting list; TTP  =  trauma treatment protocol; SMP  =  a standard stress management program.

Patients/Group characterization: T/C  =  treatment group/control group.

Cochrane tool: AA  =  adequacy of sequence allocation; AC  =  allocation concealment; BAO  =  blinding of assessors and outcomes; IO  =  incomplete outcome data; SRO  =  selective reporting and other biases.

### Assessment of Methodological Quality

The methodological quality of the studies was assessed independently by two reviewers. Eligible studies were assessed for potential bias by using the method described by the Cochrane Collaboration [17], which classified the studies as having a low, moderate, or high risk of bias across the following six domains: sequence generation, allocation concealment, blinding, missing data, selective reporting, and other biases. The research quality of the study design, patients, outcome measures, statistical analysis, and results of the selected RCTs were assessed using the approach described by Brodaty, Green, and Koschera [19], according to the guidelines established by the Cochrane Collaboration, with a total research-quality score of six to ten indicating an acceptable level of quality, and a score less than or equal to five indicating an unacceptable level of quality. Twenty-six RCTs that received a total research-quality score greater than six were included in the meta-analysis. The κ value for inter-rater reliability for the research-quality assessment was 0.89.

### Publication Bias and Sensitivity Analysis

Publication bias can influence the effect size of the outcome measures examined in meta-analyses. Publication bias in our meta-analysis was estimated using a funnel plot. An asymmetrical funnel plot indicates that supplementation is required because of missing studies [20]. The ORs reported in the selected RCTs were subjected to Egger's test [21], which uses linear regression on a natural logarithmic scale to assess funnel plot asymmetry, with the level of significance set at *p*<0.05. In addition, sensitivity analysis was performed by comparing the pooled results from the selected RCTs with those that excluded studies during selection.

### Statistical Analysis

We used the Comprehensive Meta-Analysis, Version 2.0, program for the statistical analysis. Hedges's *g* was calculated to determine the effect size [22], and Cohen's *d* [23] was calculated to obtain the overall effect size, with *d* values ≥0.8, 0.5 to 0.7, and 0.2 to 0.4 representing large, moderate, and small effect sizes, respectively. The heterogeneity among studies was determined using an *χ* ^2^-based *Q* test, with a P value>0.05 indicating a lack of heterogeneity among studies. Heterogeneity among the studies was also assessed by calculating the *I^2^* statistic, with *I^2^* values of 75%, 50%, 25%, and 0% indicating high, moderate, low, and no heterogeneity, respectively [24]. The heterogeneity data were evaluated using a random-effects model because it accommodated the possibility that the underlying effect differed across studies. The random-effects model is more conservative and has a wider 95% CI than a fixed-effects model.

### Additional Analyses

Meta-regression analysis was performed to clarify the sources of heterogeneity among the selected studies, and examine the impacts of the various exclusion criteria on the overall results. The Stata, Version 11, program (StataCorp, College Station, TX, USA) was used to perform the meta-regression analysis based on a random-effects model to determine the inherent inter-study heterogeneity in sample size, treatment duration, number of treatment sessions, year of publication, and participant characteristics, such as mean age. To understand the influence of the EMDR characteristics on the effect size and categorical variables, we used subgroup analysis to identify characteristics that led to prominent outcomes. Variables were examined using a mixed-effects model that was based on both the fixed-effects and random-effects models. Subgroup analysis was performed to determine any potential moderating variables with regard to the effect size. In the subgroup analysis, the categorical variables were evaluated using the Comprehensive Meta-Analysis, Version 2, and program. The results of group comparisons with a significant *Q_B_* indicated the potential effects of a moderator variable.

## Results

### Literature Search

As shown in Figure 1, we initially identified 1075 research reports using the search strategy. Based on the content of the title and abstracts, 333 articles were selected for further review. Among them, 304 were excluded because they described quasi-experimental studies, systematic reviews, or meta-analyses; provided no quantitative data; or used control groups that received active therapy. Of the remaining 29 RCTs, three were excluded because they were duplicate studies or involved the use of inadequate statistical analysis. The remaining 26 RCTs were included in our meta-analysis.

### Characteristics of Eligible Studies

Among the 26 RCTs selected for our study, 22, 20, 16, and 11 studies assessed the symptoms of PTSD, depression, anxiety, and subjective distress, respectively, as the primary outcome. The average age of the patients ranged from 12 to 63 years. The research-quality scores ranged from 6 to 8. Regarding the intervention, most of the studies used a manual (24 of 26), and most of them used theories (22 of 26). Most of the therapists were psychologists (14 of 26), or had group therapy experience (21 of 26; Table 1).

### Quality Assessment

The quality of the studies varied. The sequence allocation was adequate in 26 studies. Two studies [5], [9] reported allocation concealment by an independent third party. Thirteen studies [6], [9], [14], [15], [16], [25], [26], [30], [32], [34], [35], [41], [42] involved the use of blinded outcome assessors, whereas 13 studies [5], [7], [8], [10], [12], [13], [27], [28], [31], [37], [38], [39], [40] did not report blinding. Data completeness was addressed in most of the studies. Adequate assessments of each outcome and adequate selective outcome reporting were performed in all of the RCTs. Intention-to-treat analyses were conducted in three studies [7], [25], [32]. Research-quality scores of eight, seven, and six were determined for seven, twelve, and six studies, respectively.

### Publication Bias and Sensitivity Analyses

In the analysis of publication bias, the funnel plot did not appear asymmetrical, and the Egger's regression analysis of the funnel plot indicated that it was statistically symmetrical (data not shown), suggesting that publication bias did not influence our results. Sensitivity analyses were performed to assess the influence of each individual study on the pooled effect size (Hedges's *g*) based on the systematic omission of individual studies from our meta-analysis.

### Efficacy Analysis

**Figure 2:**
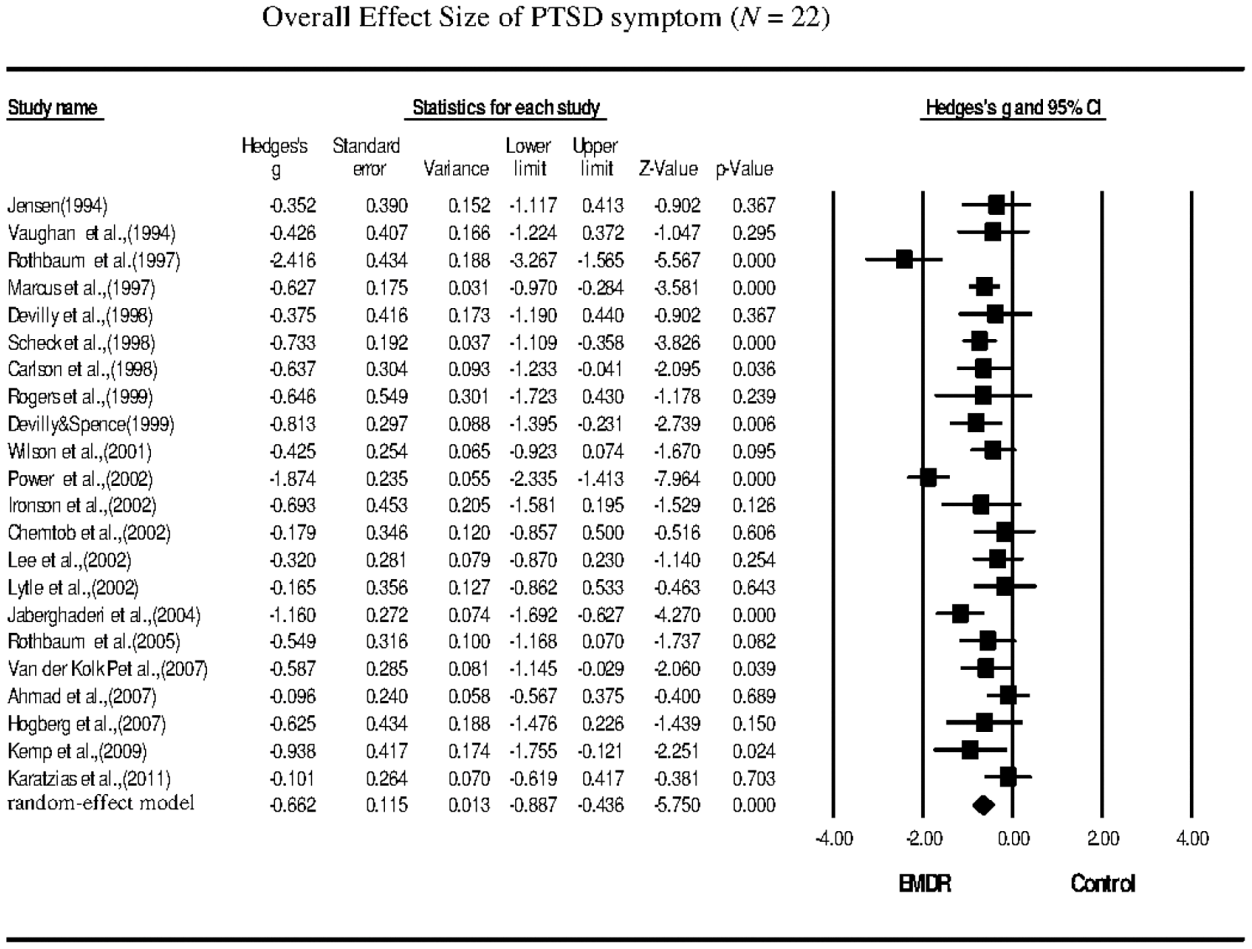
Overall effect size of the reduction in the symptoms of PTSD following EMDR therapy (*n* = 22 studies).

The effect sizes of the selected studies were significant for symptoms of PTSD, depression, anxiety, and subjective distress. The data revealed that EMDR group therapy resulted in significant improvement in patients with PTSD (Table 2). The Hedges's *g* of the 22 studies that examined PTSD symptoms following EMDR therapy was −0.662, and the 95% CI was −0.887 to −0.436 (Table 2; Figure 2). The effect sizes for sample collection were all negative, with Hedges's *g* ranging from −0.101 to −2.416. The meta-analysis revealed that the overall reduction in PTSD symptoms following EMDR therapy was significant, with a moderate effect size. Substantial heterogeneity was observed among the studies in which PTSD symptoms were the outcomes measured (*Q* = 65.062, *p* = 0.001, *I^2^* = 67.723). The funnel plot for these studies was approximately symmetrical, and the Egger's regression test revealed no publication bias (*p* = 0.98). The sensitivity analysis indicated that the removal of any one study did not affect the overall results.

**Table 2:** Overall effect size of eye movement desensitization reprocessing (EMDR) for posttraumatic stress disorder (PTSD) patients.

		Effect Size	95% CI		Null hypothesis		Homogeneity			
					(two-tailed test)					
	Sample size (studies)	Hedges's *g*	Lower	Upper	Z value	P value	Q value	P value	*I* ^2^	τ^2^
PTSD symptoms	22	−0.662	−0.887	−0.436	−5.750	0.001	65.062	0.001	67.723	0.184
Depression	20	−0.643	−0.864	−0.422	−5.713	0.001	42.657	0.001	55.458	0.133
Anxiety	16	−0.640	−0.890	−0.390	−5.025	0.001	46.804	0.001	67.951	0.164
Subjective distress	12	−0.956	−1.388	−0.525	−4.341	0.001	47.622	0.001	76.901	0.431

*P* values>0.001 were rounded to two digits.

CI, confidence interval.

**Figure 3:**
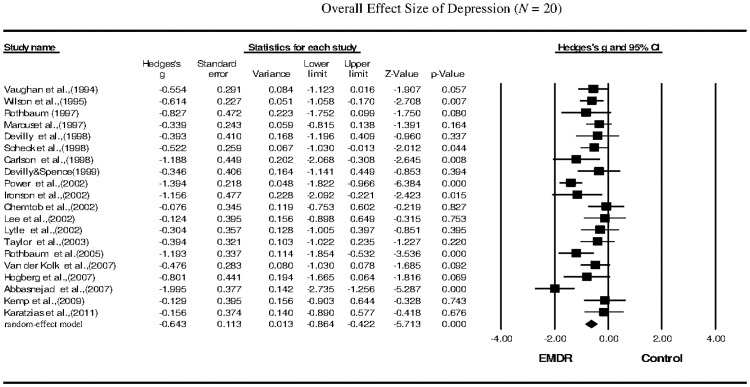
Overall effect size of the reduction in the symptoms of depression in PTSD patients following EMDR therapy (*n* = 20 studies).

Twenty studies that investigated depression as the primary outcome following EMDR therapy were included in our meta-analysis. The Hedges's *g* for the overall effect size was −0.643, and the 95% CI was −0.864 to −0.422 (Table 2, Figure 3). The effect sizes for sample collection were all negative, with the Hedges's *g* ranging from −0.076 to −1.995. These results suggested that the overall reduction in depression following EMDR therapy was significant, with a moderate effect size. Heterogeneity among the studies of depression was moderate (*Q* = 42.657, *p* = 0.001, *I^2^* = 55.458). The funnel plot for these studies was approximately symmetrical, and the Egger's regression test revealed no publication bias (*p* = 0.74). The sensitivity analysis indicated that the removal of any one study did not affect the overall results.

**Figure 4:**
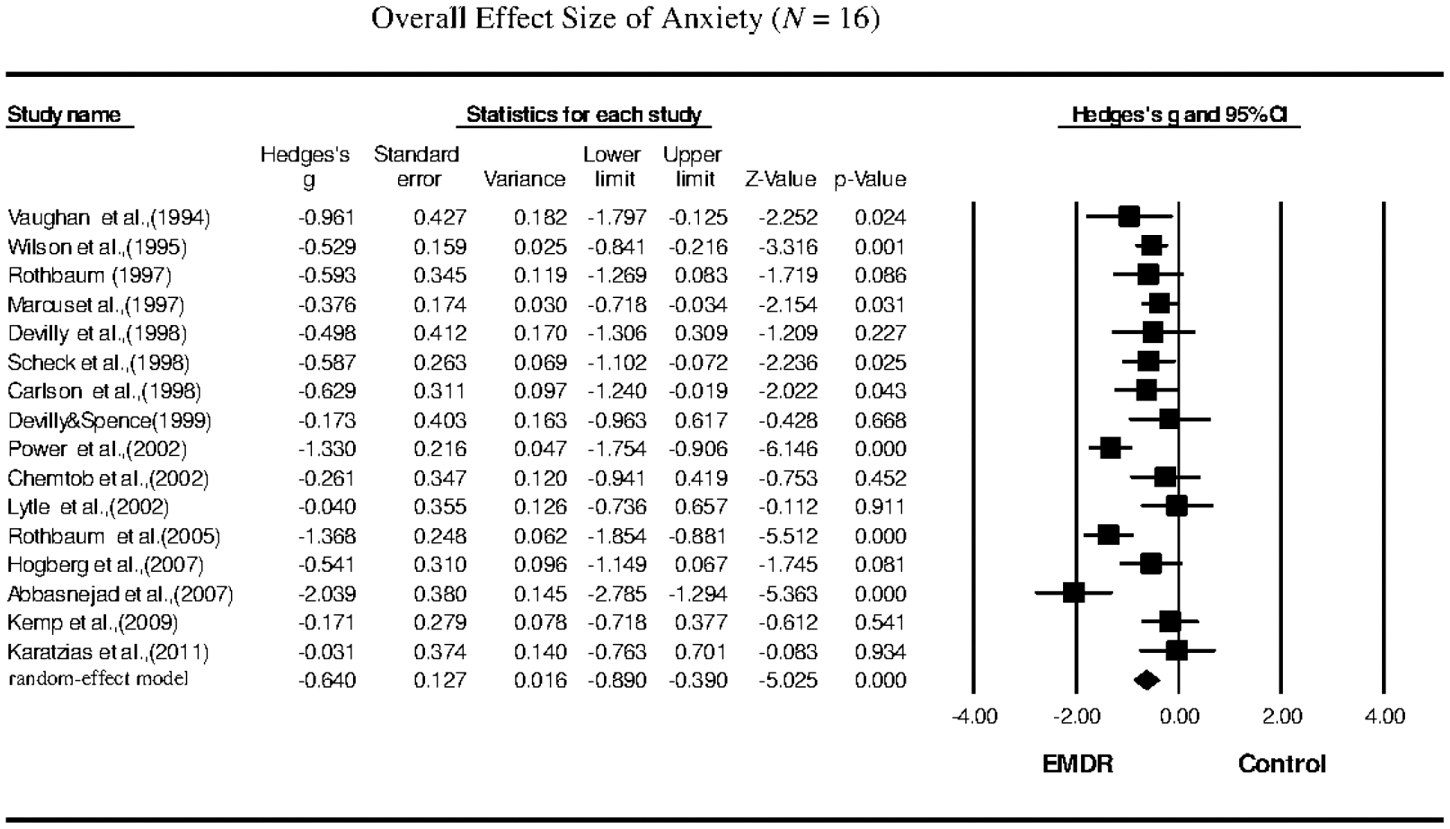
Overall effect size of the reduction in the symptoms of anxiety in PTSD patients following EMDR therapy (*n* = 16 studies).

Sixteen studies that examined anxiety as the primary outcome following EMDR therapy were included in our meta-analysis. The Hedges's *g* of the overall effect size was −0.640, with a 95% CI of −0.890 to −0.390 (Table 2; Figure 4). The effect sizes for sample collection were all negative, with Hedges's *g* ranging from −0.031 to −2.039. The results indicated that the overall reduction in anxiety following EMDR therapy was significant, with a moderate effect size. Substantial heterogeneity was observed among the anxiety studies (*Q* = 46.804, *p* = 0.001, *I^2^* = 67.951). The funnel plot for these studies was approximately symmetrical, and Egger's regression test revealed no publication bias (*p* = 0.96). The sensitivity analysis indicated that the removal of any one study did not affect the overall results.

**Figure 5:**
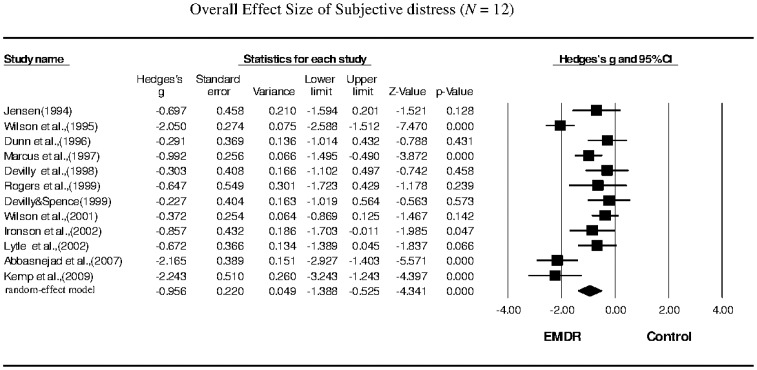
Overall effect size of the reduction in the symptoms of subjective distress in PTSD patients following EMDR therapy (*n* = 12 studies).

Twelve studies that examined subjective distress as the primary outcome were included in our meta-analysis. The Hedges's *g* for overall effect size was −0.956, with a 95% CI of −1.388 to −0.525 (Table 2; Figure 5). The effect sizes for sample collection were all negative, with Hedges's *g* ranging from −0.227 to −2.243. These results indicated that the overall improvement in subjective distress following EMDR therapy was significant, with a large effect size. Heterogeneity among the studies of subjective distress was moderate to high (*Q* = 47.622, *p* = 0.001, *I^2^* = 76.901). The funnel plot for these studies was approximately symmetrical, and the Egger's regression test revealed no publication bias (*p* = 0.93). The sensitivity analysis indicated that the removal of any one study did not affect the overall results.

### Subgroup Analyses of Posttraumatic-Stress Disorder, Depression, Anxiety, and Subjective Distress

The subgroup analysis of the improvement index for PTSD symptoms revealed that the effect size for the group led by a therapist with experience in PTSD group therapy (*g* = −0.753) was significantly larger (*Q_B_* = 7.195; *p* = 0.007) than that of the group led by a therapist without such experience (*g* = −0.234; Table 3). The subgroup analysis of the depression improvement index indicated that the effect size of a treatment duration of >60 min per session (*g* = −0.811) was significantly larger (*Q_B_* = 7.345; *p* = 0.007) than that of a treatment duration of ≤60 min per session (*g* = −0.295; Table 3). The subgroup analysis of the anxiety improvement index revealed that the effect size for a treatment duration >60 min per session (*g* = −0.860) was significantly larger (*Q_B_* = 6.191; *p* = 0.045) than that for a treatment duration ≤60 min per session (*g* = −0.351; Table 4). The subgroup analysis of the subjective distress improvement index suggested homogeneity in a majority of the studies, with only one study remaining after stratification. Thus, heterogeneity among the subjective distress studies did not appear to have influenced our results (Table 4).

**Table 3:** Subgroup analyses of posttraumatic stress disorder (PTSD) symptoms and depression.

	PTSD symptoms					Depression					
	Sample size (studies)	Hedges's *g* (95% CI)	*P_A_*	*Q_B_*	*P_B_*	Sample size (studies)		Hedges's *g* (95% CI)	*P_A_*	*Q_B_*	*P_B_*
		**Therapy characteristics**				**Therapy characteristics**					
**Manual**											
Yes	21	−0.667 (−0.903, −0.431)	0.001	0.123	0.726	18		−0.550 (−0.742, −0.359)	0.001	6.185	0.013
No/Undisclosed	1	−0.549 (−1.168, −0.070)	0.082			2		−1.576 (−2.362, −0.791)	0.001		
**Shapiro**											
Yes	20	−0.689 (−0.933, −0.445)	0.001	1.119	0.290	17		−0.692 (−0.951, −0.433)	0.001	1.278	0.258
No/Undisclosed	2	−0.422 (−0.853, 0.009)	0.055			3		−0.459 (−0.768, −0.151)	0.004		
**Instrument**											
self-rating scales	12	−0.786(−1.060, −0.511)	0.001	1.400	0.237	18		−0.642(−0.886, −0398)	0.001	0.002	0.962
non-self-rating scales	10	−0.520(−0.864, −0.176)	0.003			2		−0.629(−1.104, −0.153)	0.010		
**Treatment duration**											
≤60 min	10	−0.503 (−0.741, −0.265)	0.001	3.707	0.157	6		−0.295 (−0.551, −0.038)	0.024	7.345	0.007
>60 min	11	−0.844 (−1.214, −0.475)	0.001			14		−0.811 (−1.082, −0.539)	0.001		
Undisclosed	1	−0.179 (−0.857, 0.500)	0.606								
**Treatment sessions**											
<3	5	−0.800 (−1.467, −0.132)	0.019	0.308	0.579	4		−0.700 (−1.271, −0.128)	0.016	0.068	0.794
≥3	17	−0.601 (−0.816, −0.386)	0.001			16		−0.617 (−0.855, −0.380)	0.001		
**Control type**											
Waiting list group	9	−0.835(−1.364, −0.305)	0.002	0.840	0.359	9		−0.839(−1.233, −0.446)	0.001	2.939	0.086
Equivalent group	13	−0.577(−0.731, −0.422)	0.001			11		−0.453(−0.653, −0.253)	0.001		
		**Therapist characteristics**						**Therapist characteristics**			
**Therapist background**											
Psychologist	17	−0.660 (−0.894, −0.425)	0.001	0.036	0.849	17		−0.548 (−0.754, −0.343)	0.001	2.480	0.115
Nonpsychologist	5	−0.727 (−1.381, −0.073)	0.029			3		−1.220 (−2.031, −0.085)	0.003		
**PTSD group experience**											
Yes	18	−0.753 (−1.005, −0.502)	0.000	7.195	0.007	17		−0.643 (−0.890, −0.395)	0.001	0.001	0.992
No/Undisclosed	4	−0.234 (−0.518, −0.049)	0.106			3		−0.646 (−1.206, −0.258)	0.002		
	**Participant characteristics**					**Participant characteristics**					
**Age**											
Children/adolescents (6–17 y)	4	−0.578 (−1.148, −0.007)	0.047	0.106	0.744	3	−0.731 (−1.964, 0.501)		0.245	0.023	0.878
Adults (18–64 y)	18	−0.681 (−0.934, −0.429)	0.001			17	−0.634 (−0.831, −0.436)		0.001		

*P_A_*, subgroup effect on outcome variable; *P_B_*, heterogeneity among subgroups (moderator); CI, confidence interval.

**Table 4:** Subgroup analyses of anxiety and subjective distress.

	Anxiety						Subjective distress					
	Sample size (studies)		Hedges' s *g* (95% CI)	*P_A_*	*Q_B_*	*P_B_*	Sample size (studies)		Hedges's *g* (95% CI)	*P_A_*	*Q_B_*	*P_B_*
	**Therapy characteristics**						**Therapy characteristics**					
**Manual**												
Yes	14	−0.511 (−0.709, −0.313)		0.001	10.731	0.00101	11		−0.847 (−1.265, −0.429)	0.001	8.841	0.003
No/Undisclosed	2	−1.642 (−2.289, −0.995)		0.001			1		−2.165 (−2.927, −1.403)	0.001		
**Shapiro**												
Yes	13	−0.590 (−0.853, −0.327)		0.001	0.439	0.508	10		−0.683 (−0.995, −0.372)	0.001	26.147	0.001
No/Undisclosed	3	−0.909 (−1.813, −0.005)		0.049			2		−2.088 (−2.528, −1.649)	0.001		
**Instrument**												
self-rating scales	15	−0.646(−0.911, −0.381)		0.001	0.097	0.756	12		−0.956(−1.388, −0.525)	0.001	47.622	0.001
non-self-rating scales	1	−0.541(−1.149, 0.067)		0.081			0					
**Treatment duration**												
≤60 min	6	−0.351 (−0.575, −0.127)		0.002	6,191	0.045	4		−1.083 (−1.664, −0.503)	0.001	3.017	0.221
>60 min	9	−0.860 (−1.210, −0.509)		0.001			7		−0.962 (−1.634, −0.291)	0.005		
Undisclosed	1	−0.261 (−0.941, 0.419)		0.452			1		−0.291 (−1.014, 0.432)	0.431		
**Treatment sessions**												
<3	4	−0.659 (−1.240, −0.077)		0.026	0.009	0.925	3		−0.535 (−1.013, −0.057)	0.028	4.506	0.105
≥3	12	−0.628 (−0.911, −0.344)		0.001			8		−1.186 (−1.754, −0.619)	0.001		
Undisclosed							1		−0.291 (−1.014, 0.432)			
**Control type**												
Waiting list group	8	−0.845(−1.249, −0.441)		0.001	3.503	0.061	4		−1.814(−2.453, −1.176)	0.001	12.729	0.001
Equivalent group	8	−0.412(−0.618, −0.205)		0.001			8		−0.572(−0.813, −0.332)	0.001		
		**Therapist characteristics**							**Therapist characteristics**			
**Therapist background**												
Psychologist	13	−0.490 (−0.694, −0.286)		0.001	10.933	0.001	9		−0.977 (−1.454, −0.499)	0.001	0.008	0.929
Nonpsychologist	3	−1.462 (−2.001, −0.923)		0.001			3		−0.922 (−2.024, 0.181)	0.101		
**PTSD group experience**												
Yes	13	−0.598 (−0.857, −0.339)		0.001	0.240	0.624	11		−1.019 (−1.474, −0.564)	0.001	2.789	0.095
No/Undisclosed	3	−0.814 (−1.637, −0.009)		0.053			1		−0.291 (−1.014, 0.432)	0.431		
	**Participant characteristics**						**Participant characteristics**					
**Age**												
Children/adolescents (6–17 y)	3	−0.804 (−1.922, 0.314)		0.159	0.097	0.755	1	−2.243 (−3.243, −1.243)		0.001	6.200	0.013
Adults (18–64 y)	13	−0.622 (−0.862, −0.383)		0.001			11	−0.861 (−1.290, −0.432)		0.001		

*P_A_*, subgroup effect on outcome variable; *P_B_*, heterogeneity among subgroups (moderator); CI, confidence in.

### Meta-Regression Analyses

The meta-analyses of the RCTs conducted to investigate the effects of EMDR on the symptoms of PTSD, depression, anxiety, and subjective distress in PTSD patients were performed using unrestricted maximum-likelihood meta-regressions. No significant relationship was observed between the effect size for PTSD and participant age (*β* = −0.01, *t* = −0.69), publication year (*β* = 0.02, *t* = 1.00), sample size (*β* = −0.01, *t* = −0.41), the number of treatment sessions (*β* = 0.03, *t* = 1.02), or treatment duration (*β* = −0.01, *t* = −1.10). No significant relationship was observed between the effect size for depression and participant age (*β* = −0.02, *t* = −1.78), publication year (*β* = 0.01, *t* = 0.98), sample size (*β* = −0.01, *t* = −0.43), number of treatment sessions (*β* = −0.01, *t* = −0.13), or treatment duration (*β* = −0.01, *t* = −1.45). No significant relationship was observed between the effect size for anxiety and participant age (*β* = −0.01, *t* = −1.37), publication year (*β* = 0.01, *t* = 0.10), sample size (*β* = −0.01, *t* = −1.35), number of treatment sessions (*β* = −0.01, *t* = −0.10), or treatment duration (*β* = −0.01, *t* = −1.45). No significant relationship was observed between the effect size for subjective distress and participant age (*β* = 0.03, *t* = 1.38), publication year (*β* = −0.08, *t* = −1.53), sample size (*β* = −0.01, *t* = −0.81), number of treatment sessions (*β* = 0.06, *t* = 0.23), or treatment duration (*β* = 0.01, *t* = 0.34).

## Discussion

### Main Findings

The objective of the current study was to perform meta-analysis on previously reported RCTs to determine the magnitude of the effects of EMDR therapy on the symptoms of PTSD, depression, anxiety, and subjective distress in patients with PTSD. The meta-analysis revealed that the effect sizes for EMDR therapy for PTSD, depression, and anxiety were moderate, whereas the effect size for EMDR therapy for subjective distress was large. These results suggest that EMDR therapy can improve self-awareness in patients, change their beliefs and behaviors, reduce anxiety and depression, and lead to positive emotions.

PTSD patients cannot properly manage their negative experiences or memories. EMDR therapy involves the use of eye movements to induce ORs, and enables PTSD patients to create adaptive connections to integrate negative experiences with positive emotions and cognitions, thereby significantly improving PTSD symptoms. Our findings were similar to those of Davidson and Parker [3], who conducted a meta-analysis on quantitative studies of EMDR therapy published between 1988 and 2000. They reported a moderate effect size for EMDR therapy (*r* = 0.40, Cohen's *d* = 0.87), compared with that of other non-specified therapies. Other studies have reported that EMDR therapy produced increased reductions in PTSD symptoms, compared with that produced by medication therapy and control groups [6], [32].

Depression is often comorbid with PTSD [33]. Twenty of the 26 studies included in our meta-analysis indicated that EMDR therapy significantly reduced the symptoms of depression in patients with PTSD, and our analysis revealed a moderate effect size for EMDR therapy for depression. Our findings are consistent with those of other studies on depression, which have demonstrated that EMDR group therapy significantly reduced the symptoms of depression, compared with control groups [5], [7], [12], [30].

Specific traumatic stressors cause PTSD patients to experience anxiety when coping with stress. Sixteen of the 26 studies included in our meta-analysis reported that EMDR therapy significantly reduced anxiety in PTSD patients, and our analysis revealed a moderate effect size for EMDR therapy for anxiety. Our findings are consistent with those of Scheck et al. [9], who reported that EMDR therapy significantly reduced anxiety in women with PTSD, with a moderate effect size (Cohen's *d* = 0.66). Our findings are also consistent with those of Abbasnejad et al. [8] and Power et al. [6], which indicated that EMDR therapy significantly reduced the symptoms of anxiety in patients, compared with those experienced by control patients awaiting treatment. EMDR therapy relieves anxiety by reprocessing information when PTSD patients undergo a subsequent traumatic event.

Patients with PTSD experience subjective distress because they have been disturbed by previous negative experiences, and wish to avoid the memories of those experiences. Our meta-analysis of 12 studies on the effects of EMDR therapy on subjective distress revealed that the effect size was large. Wilson et al. [16] examined the effects of EMDR therapy on 62 police officers who had experienced traumatic events, and their results indicated that EMDR therapy significantly reduced subjective distress, with a large effect size (Cohen's *d* = 2.07). Our findings are consistent with those of a meta-analysis conducted by Davidson and Parker in 2001 [3], which included quantitative studies related to EMDR therapy published between 1988 and 2000. They reported that EMDR therapy reduced subjective distress, with a large effect size (*r* = 0.81, Cohen's *d* = 2.71). Kemp et al. [12] also demonstrated that, compared with a control group awaiting treatment, subjective distress was significantly reduced in patients who had undergone EMDR therapy. Thus, EMDR therapy reduces anxiety and subjective distress when patients undergo a subsequent traumatic event.

### Subgroup Findings

We performed subgroup analysis based on the characteristics of the therapist, the intervention, and the study design and methodology. Treatment duration per session was the principal characteristic of the intervention. Our subgroup analysis indicated that a treatment duration of >60 min per session was more effective than shorter treatment durations, which significantly reduced both anxiety and depression. Previous studies on EMDR therapy involving the use of interventions ranging from 50 to 120 min in duration have reported reduced PTSD symptoms, but the levels of improvement were inconsistent [12], [13], [16], [30], [34], [35], suggesting that a potential moderator influenced the effects of EMDR therapy. Nonetheless, these findings are consistent with those of our meta-analysis, which revealed that a treatment duration of >60 min per session reduced anxiety and depression in patients with PTSD.

We discovered that patients exhibited greater reductions in PTSD symptoms when they received EMDR therapy from therapists experienced in PTSD-group therapy, compared with those treated by therapists without such experience. This subgroup analysis result also reflects the benefits of EMDR therapy for PTSD patients. These findings are generally consistent with those of previous studies [29], [36], which have reported that patients exhibited greater reductions in the symptoms of depression following cognitive therapy when treated by a therapist experienced in group therapy, compared with those treated by therapists without group-therapy experience.

### Limitations

The major limitation of the present study is that considerable variations were observed in the study designs, outcome measurement scales, and sample sizes of the various RCTs included in our meta-analysis, which affected the overall effect size and the results of the overall subgroup analysis. The accuracy of the effect size estimation also affected the meta-analysis results. Furthermore, the methods of data collection were inconsistent among the various RCTs selected, and the details of data collection were provided only in studies in which intention-to-treat analyses were performed, which might have led to an overestimation of the effect size. By contrast, missing baseline values might have led to an underestimation of the effect size.

### Implications

Our meta-analysis of RCTs revealed that EMDR may be helpful for treating PTSD and depression, anxiety, and subjective distress in PTSD patients. We determined that therapists experienced in PTSD group therapy and a duration of treatment >60 min per session also contributed to reductions in the symptoms of PTSD, depression, anxiety, and subjective distress in PTSD patients following EMDR therapy. In addition, the effect sizes determined in our meta-analysis support EMDR as the optimal type of psychotherapy for PTSD patients.

## Supporting Information

Checklist S1 PRISMA Checklist.
(PDF)

## References

[1] Shapiro F, Solomon RM (1995) Eye movement desensitization and reprocessing: Wiley Online Library.

[2] Shapiro F (2001) Eye movement desensitization and reprocessing: Basic principles, protocols, and procedures, 2nd ed. New York: The Guilford Press.

[3] Davidson PR, Parker KC (2001) Eye movement desensitization and reprocessing (EMDR): a meta-analysis. J Consult Clin Psychol 69: 305–316. 11393607 10.1037//0022-006x.69.2.305

[4] Rodenburg R, Benjamin A, de Roos C, Meijer AM, Stams GJ (2009) Efficacy of EMDR in children: a meta-analysis. Clin Psychol Rev 29: 599–606. 19616353 10.1016/j.cpr.2009.06.008

[5] Hogberg G, Pagani M, Sundin O, Soares J, Aberg-Wistedt A, et al (2007) On treatment with eye movement desensitization and reprocessing of chronic posttraumatic stress disorder in public transportation workers—a randomized controlled trial. Nord J Psychiatry 61: 54–61. 17365790 10.1080/08039480601129408

[6] Power K, McGoldrick T, Brown K, Buchanan R, Sharp D, et al (2002) A controlled comparison of eye movement desensitization and reprocessing versus exposure plus cognitive restructuring versus waiting list in the treatment of posttraumatic stress disorder. Clinical Psychology & Psychotherapy 9: 299–318.

[7] Rothbaum BO, Astin MC, Marsteller F (2005) Prolonged Exposure versus Eye Movement Desensitization and Reprocessing (EMDR) for PTSD rape victims. J Trauma Stress 18: 607–616. 16382428 10.1002/jts.20069

[8] Abbasnejad MMK, Zamyad A (2007) Efficacy of eye movement desensitization and reprocessing in reducing anxiety and unpleasant feelings due to earthquake experience. Psychol Res 9: 104–117.

[9] Scheck MM, Schaeffer JA, Gillette C (1998) Brief psychological intervention with traumatized young women: the efficacy of eye movement desensitization and reprocessing. J Trauma Stress 11: 25–44. 9479674 10.1023/A:1024400931106

[10] Dunn TM, Schwartz M, Hatfield RW, Wiegele M (1996) Measuring effectiveness of eye movement desensitization and reprocessing (EMDR) in non-clinical anxiety: a multi-subject, yoked-control design. J Behav Ther Exp Psychiatry 27: 231–239. 8959424 10.1016/s0005-7916(96)00034-1

[11] Grainger RD, Levin C, Allen-Byrd L, Doctor RM, Lee H (1997) An empirical evaluation of eye movement desensitization and reprocessing (EMDR) with survivors of a natural disaster. J Trauma Stress 10: 665–671. 9391949 10.1023/a:1024806105473

[12] Kemp M, Drummond P, McDermott B (2010) A wait-list controlled pilot study of eye movement desensitization and reprocessing (EMDR) for children with posttraumatic stress disorder (PTSD) symptoms from motor vehicle accidents. Clin Child Psychol Psychiatry 15: 5–25. 19923161 10.1177/1359104509339086

[13] Lytle RA, Hazlett-Stevens H, Borkovec TD (2002) Efficacy of Eye Movement Desensitization in the treatment of cognitive intrusions related to a past stressful event. J Anxiety Disord 16: 273–288. 12214813 10.1016/s0887-6185(02)00099-3

[14] Marcus SVMP, Sakai C (1997) Controlled study of treatment of PTSD using EMDR in an HMO setting. Psychotherapy: Theory, Research, Practice, Training 34: 307.

[15] Rogers S, Silver SM, Goss J, Obenchain J, Willis A, et al (1999) A single session, group study of exposure and Eye Movement Desensitization and Reprocessing in treating Posttraumatic Stress Disorder among Vietnam War veterans: preliminary data. J Anxiety Disord 13: 119–130. 10225504 10.1016/s0887-6185(98)00043-7

[16] Wilson S, Tinker R, Becker L, Logan C (2001) Stress Management with Law Enforcement Personnel: A Controlled Outcome Study of EMDR Versus a Traditional Stress Management Program. International Journal of Stress Management 8: 179–200.

[17] Higgins JPTGS (2008) Cochrane Handbook for Systematic Reviews of Interventions Version 5.0.1. The Cochrane Collaboration.

[18] Cohen J (1960) A coefficient of agreement for nominal scales. Educational and psychological measurement 20: 37–46.

[19] Brodaty H, Green A, Koschera A (2003) Meta-analysis of psychosocial interventions for caregivers of patients with dementia. J Am Geriatr Soc 51: 657–664. 12752841 10.1034/j.1600-0579.2003.00210.x

[20] Richard J (1984) Summing up: the science of reviewing research: Harvard University Press.

[21] Egger M, Davey Smith G, Schneider M, Minder C (1987) Bias in meta-analysis detected by a simple, graphical test. BMJ 315: 629–634. 10.1136/bmj.315.7109.629 PMC2127453 9310563

[22] Borenstein M, Hedges LV, Rothstein H (2007) Introduction to meta-analysis. Chichester, England: Wiley.

[23] Cohen J (1988) Statistical Power Analysis for the Behavioral Sciences: L. Erlbaum Associates.

[24] Higgins JP, Thompson SG, Deeks JJ, Altman DG (2003) Measuring inconsistency in meta-analyses. Bmj 327: 557–560. 12958120 10.1136/bmj.327.7414.557 PMC192859

[25] Ahmad A, Larsson B, Sundelin-Wahlsten V (2007) EMDR treatment for children with PTSD: results of a randomized controlled trial. Nord J Psychiatry 61: 349–354. 17990196 10.1080/08039480701643464

[26] Carlson JG, Chemtob CM, Rusnak K, Hedlund NL, Muraoka MY (1998) Eye movement desensitization and reprocessing (EDMR) treatment for combat-related posttraumatic stress disorder. J Trauma Stress 11: 3–24. 9479673 10.1023/A:1024448814268

[27] Devilly GJ, Spence SH (1999) The relative efficacy and treatment distress of EMDR and a cognitive-behavior trauma treatment protocol in the amelioration of posttraumatic stress disorder. J Anxiety Disord 13: 131–157. 10225505 10.1016/s0887-6185(98)00044-9

[28] Devilly GJSS, Rapee RM (1998) The relative efficacy and treatment distress of EMDR and a cognitive-behavior trauma treatment protocol in the amelioration of posttraumatic stress disorder. Journal of anxiety disorders 29: 435–455. 10.1016/s0887-6185(98)00044-9 10225505

[29] Haby MM, Donnelly M, Corry J, Vos T (2006) Cognitive behavioural therapy for depression, panic disorder and generalized anxiety disorder: a meta-regression of factors that may predict outcome. Aust N Z J Psychiatry 40: 9–19. 16403033 10.1080/j.1440-1614.2006.01736.x

[30] Karatzias T, Power K, Brown K, McGoldrick T, Begum M, et al (2011) A controlled comparison of the effectiveness and efficiency of two psychological therapies for posttraumatic stress disorder: eye movement desensitization and reprocessing vs. emotional freedom techniques. J Nerv Ment Dis 199: 372–378. 21629014 10.1097/NMD.0b013e31821cd262

[31] Lee C, Gavriel H, Drummond P, Richards J, Greenwald R (2002) Treatment of PTSD: stress inoculation training with prolonged exposure compared to EMDR. J Clin Psychol 58: 1071–1089. 12209866 10.1002/jclp.10039

[32] van der Kolk BA, Spinazzola J, Blaustein ME, Hopper JW, Hopper EK, et al (2007) A randomized clinical trial of eye movement desensitization and reprocessing (EMDR), fluoxetine, and pill placebo in the treatment of posttraumatic stress disorder: treatment effects and long-term maintenance. J Clin Psychiatry 68: 37–46. 17284128 10.4088/jcp.v68n0105

[33] Bisson JI (2007) Posttraumatic stress disorder. Occupational Medicine 57: 399–403. 17728312 10.1093/occmed/kqm069

[34] Rothbaum BO (1997) A controlled study of eye movement desensitization and reprocessing in the treatment of posttraumatic stress disordered sexual assault victims. Bull Menninger Clin 61: 317–334. 9260344

[35] Vaughan K, Armstrong MS, Gold R, O'Connor N, Jenneke W, et al (1994) A trial of eye movement desensitization compared to image habituation training and applied muscle relaxation in posttraumatic stress disorder. J Behav Ther Exp Psychiatry 25: 283–291. 7706505 10.1016/0005-7916(94)90036-1

[36] DeRubeis RJ, Hollon SD, Amsterdam JD, Shelton RC, Young PR, et al (2005) Cognitive therapy vs medications in the treatment of moderate to severe depression. Arch Gen Psychiatry 62: 409–416. 15809408 10.1001/archpsyc.62.4.409

[37] Jensen JA (1994) An investigation of eye movement desensitization and reprocessing (EMD/R) as a treatment for posttraumatic stress disorder (PTSD) symptoms of Vietnam combat veterans. Behavior Therapy 25: 311–325.

[38] Wilson SA, Becker LA, Tinker RH (1995) Eye movement desensitization and reprocessing (EMDR) treatment for psychologically traumatized individuals. J Consult Clin Psychol 63: 928–937. 8543715 10.1037//0022-006x.63.6.928

[39] Ironson G, Freund B, Strauss JL, Williams J (2002) Comparison of two treatments for traumatic stress: a community-based study of EMDR and prolonged exposure. J Clin Psychol 58: 113–128. 11748600 10.1002/jclp.1132

[40] Chemtob CM, Nakashima J, Carlson JG (2002) Brief treatment for elementary school children with disaster-related posttraumatic stress disorder: a field study. J Clin Psychol 58: 99–112. 11748599 10.1002/jclp.1131

[41] Taylor S, Thordarson DS, Maxfield L, Fedoroff IC, Lovell K, et al (2003) Comparative efficacy, speed, and adverse effects of three PTSD treatments: exposure therapy, EMDR, and relaxation training. J Consult Clin Psychol 71: 330–338. 12699027 10.1037/0022-006x.71.2.330

[42] Jaberghaderi N, Greenwald R, Rubin A, Zand SO, Dolatabadi S (2004) A comparison of CBT and EMDR for sexually-abused Iranian girls. Clinical Psychology & Psychotherapy 11: 358–368.

